# Effect of Delayed Diagnosis of Phenylketonuria With Imaging Findings of Bilateral Diffuse Symmetric White Matter Lesions: A Case Report and Literature Review

**DOI:** 10.3389/fneur.2019.01040

**Published:** 2019-10-04

**Authors:** Shuna Chen, Mingqin Zhu, Yulei Hao, Jiachun Feng, Ying Zhang

**Affiliations:** Department of Neurology and Neuroscience Center, The First Hospital of Jilin University, Changchun, China

**Keywords:** neurogenetics, phenylketonuria, leukodystrophy, inherited vasculopathy, mitochondrial disorders, adult-onset, late-diagnoed PKU

## Abstract

Phenylketonuria is a hereditary metabolic disorder due to the deficiency of tetrahydrobiopterin or phenylalanine hydroxylase. Delayed diagnoses of it manifest a progressive irreversible neurological impairment in the early years of the disease. Guthrie test and tandem mass spectrometry aided in early detection and intervention of phenylketonuria, which significantly decreased the disability of patients as well as reducing the need for diagnosis in adults. This is a case report of a 60-year-old Asian man, characterized by severe visual-spatial disorders and bilateral diffuse symmetric white matter lesions on magnetic resonance imaging, who was diagnosed as phenylketonuria with his congenital mental retardation sibling. Heterozygous mutations exist in gene encoding PAH c.1068C>A and c.740G>T. During the diagnosis, we looked up at other late-onset genetic diseases considered to occur rarely but gradually revealed similar clinical manifestations and significant white matter lesions gaining importance in guiding to correct diagnosis and treatment. We made a comprehensive review of phenylketonuria and other inherited diseases with major prevalence in adulthood with prominent white matter involvement. Our study aims to help neurologists to improve recognition of metabolism-related leukoencephalopathies without neglect of the role of congenital genetic factors.

## Introduction

**Figure 1:**
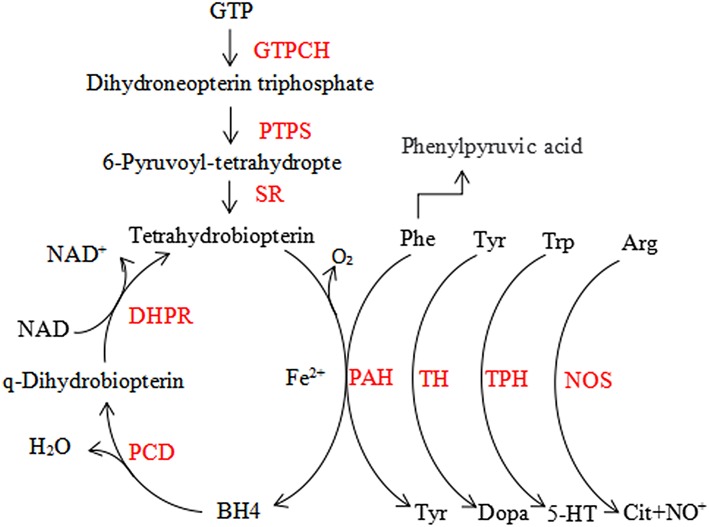
The phenylalanine hydroxylating system. Phe, phenylalanin; Tyr, tyrosine; Trp, tryptophan; 5-HT, serotonin; Arg, arginine; Cit, citrulline; BH4, tetrahydrobiopterin; GTPCH, GTP cyclohydrolase I; PTPS, 6-pyruvoyl-tetrahydropterin synthase; SR, sepiapterin reductase; DHPR, dihydropteridine reductase; PCD, pterin-4a-carbinolamine dehydratase; PAH, phenylalanine hydroxylase; TH, tyrosine hydroxylase; TPH, tryptophan hydroxylase; NOS, nitric oxide synthase.

Phenylketonuria (PKU) is the most prevalent disorder caused by an inborn error in amino acid metabolism, but it is curable. The prevalence of it varies widely around the world (1). PKU is characterized by phenylalanine (Phe) accumulation mostly due to hepatic phenylalanine hydroxylase (PAH) deficiency, which converts Phe to tyrosine (Try), requiring the cofactor tetrahydrobiopterin (BH4), molecular oxygen and iron (1). BH4 is the essential cofactor for PAH, as well as for the metabolism of catecholamines, serotonin, and nitric oxide in the central nervous system (CNS) (Figure 1) (2). Clinical findings report that the deficiency of BH4 metabolism due to hereditary accounts for about 1–2% among the patients with hyperphenylalaninemia (HPA), which is more severe compared to PKU (3). Newborn children are routinely screened for PKU, but mass spectrometry (MS) was used in the countries with expanded newborn screening to diagnose it, and for positive test results Phe value confirmation is must. Internationally accepted Phe cut-off level for PKU diagnosis is 120 μM (with a Phe/Tyr ratio >2) (4). It is important to exclude BH4 deficiency in infants, even if they have mild HPA, to prevent further progression which may cause severe harm to the CNS (5). The 2012 National Institute of Health (US) PKU conference (6) classified patients as follows, based on the peak blood Phe concentration without treatment: (1) mild hyperphenylalaninemia (MHP) [(Phe): 120–360 μM]; (2) mild HPA-gray zone [(Phe): 360–600 μM]; (3) mild PKU [(Phe): 600–900 μM]; (IV) moderate PKU [(Phe): 900–1,200 μM]; (4) and classic PKU (cPKU) [(Phe): >1,200 μM]. Those in Blau (3), Blau et al. (4), and Blau et al. (5) must be treated. However, as per the European guidelines in 2017, even patients with Phe mentioned in Werner et al. (2) are advised for treatment (7). The basis for PKU treatment is a low Phe diet. Few can take advantage from BH4 (8), large neutral amino acids (LNAA) (9), casein glycomacropeptide (10), Phenylalanine ammonia lyase (11), and gene therapy (12). We targeted Phe blood concentration of 360, 600 μM as the upper limit for the first 12 years of life and for individuals older than 12 years, respectively (13). Patients who are subjected to a strict Phe reduced diet after birth will develop a normal intellectual and neurological system, while delayed diagnoses and untreated PKU develops into severe neurological outcome such as microcephaly, mental retardation, epilepsy, and else. In rare cases, the first sign of PKU develops in the late adulthood resembling common manifestations of neurological diseases such as progressive dementia, spastic paraplegia, ataxia, tremor, and behavioral problems.

In this case report, we present a 60-year-old Asian man diagnosed as PKU, and whose clinical features and brain MRI indicated severe CNS damage with significantly elevated Phe levels (1221.5μM, Phe/Tyr ratio 27.45) in blood. We also summarized the demographic and medical characteristics of patients published on PubMed database between January 1993 and March 2019 related to data of adult-onset or delayed diagnosed PKU in Supplementary Table 1 along with our case. Further, we listed the main characteristics of other hereditary leukoencephalopathies in adulthood in Supplementary Tables 2, 3.

## Case Report

**Figure 2:**
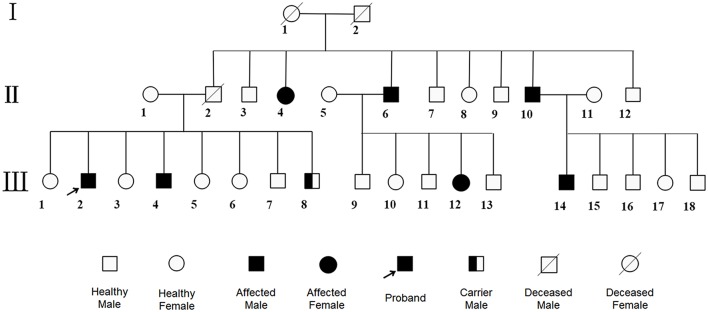
Pedigree. The patient and his fourth brother showed heterozygous mutations in exon12 encoding PAH c.1068C>A and c.740G>T in WES, while the youngest brother was a carrier with only c.1068C>A. Other family members were roughly identified by positive clinical manifestations. No consanguineous marriage in this family.

A 60-year-old Chinese Han single man got admitted to our hospital for 2 months of visual-spatial impairment and personality change. He developed visual orientation disorders and couldn't walk with stability in 2 months, which aggravated, gradually stopping him from leading an independent life. It was further accompanied by fumbling behavior and he became unresponsive to external stimuli. The patient reported having excessive sweat, difficult urination, and constipation, but did not show significant weight loss. He lived in a village in Northern China, where the main diet was pasta. He had convulsions at the age of three and, since then, he began to show cognitive impairment which was evident since he was not able to count beyond two numbers. Never been to school, he lived on his own performing some simple farming work under the supervision of his younger brother. He had no history of smoking, drinking, and toxic exposure. Family medical history revealed six of his family members suffered from mental retardation (Figure 2). Physical examination showed tan pupils and fair sweaty skin. Neurological examinations were characterized by blurred consciousness, decreased eye blink, absent eyeball movement, spastic paraparesis with tremor, brisk tendon reflexes, bilateral pyramidal sign. The in-depth analysis could not be done as the patient did not extend support.

Routine blood tests revealed the homocysteine (Hcy) level as 90.2 μM (normal 0–20 μM), vitamin B12 level as 72.00 pM (normal 133–675 pM), and aspartate transaminase as 46.9 U/L (normal 13–35 U/L). Renal function, electrolyte, uric acid, trace elements, folate, blood adrenocorticotropic hormone level, thyroid function, HIV antibody, tumor makers, hepatitis B test, rapid plasma regain test were obtained and found to be normal.

**Figure 3:**
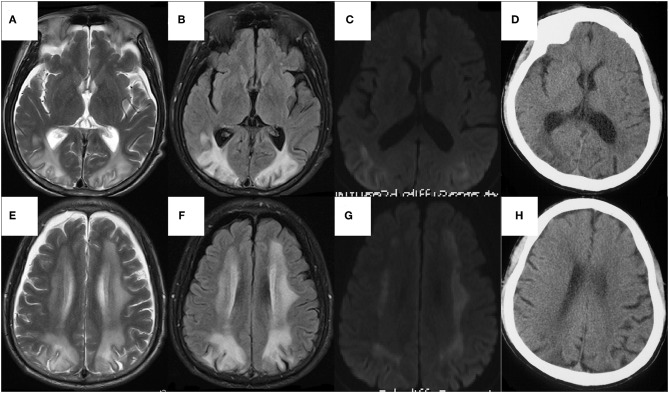
The axial sequences on head MRI of the patient **(A–F)** and The head CT of the sick brother **(G,H)**. **(A–F)** Bilateral symmetric diffuse white matter hyperintensity, predominantly involving the frontal, parietal, occipital, and periventricular regions on T2WI **(A,B)** and FLAIR **(C,D)**, with restriction in peripheral areas of lesions on DWI **(E,F)**, whereas normal on ADC map. The U-fibers of bilateral occipital lobe and the white matter of paracortical, subcortical were involved but corpus callosum and internal capsule were relatively reserved. There was atrophy in temporal and frontal lobes, especially significant in frontal pole. **(G,H)** The low-density lesions around bilateral ventricles mainly involving white matter around the posterior horn of lateral ventricles, with atrophy of temporal and frontal lobes especially in frontal pole.

We performed lumbar puncture on patients and the relative results showed that intracranial pressure was 190 mm H_2_O, protein was 0.89g/L (normal 0.15–0.45 g/L), CSF immunoglobulin G was 98.2 mg/L (normal 0–34 mg/L) with normal glucose and cells. An electroencephalogram represented general slowing and irregular slow waves. Doppler ultrasound exhibited bilateral multiple plaques of carotid and the initial segment of the right internal carotid artery with mild stenosis (<50%). The axial T2-weighted magnetic resonance imaging (T2WI) and Fluid Attenuated Inversion Recovery (FLAIR) sequences on brain MRI revealed bilateral symmetric diffuse white matter hyperintensity, predominantly involving the frontal, parietal, occipital, and periventricular regions with restriction in peripheral areas of lesions on diffusion weighted imaging (DWI) (Figures 3A–C,E–G). We managed to get the head CT of the patient's sick brother concurrently and the results suggested the existence of low-density lesions in the bilateral frontal and posterior horn of lateral ventricle but comparably milder than patient (Figures 3D,H).

**Figure 4:**
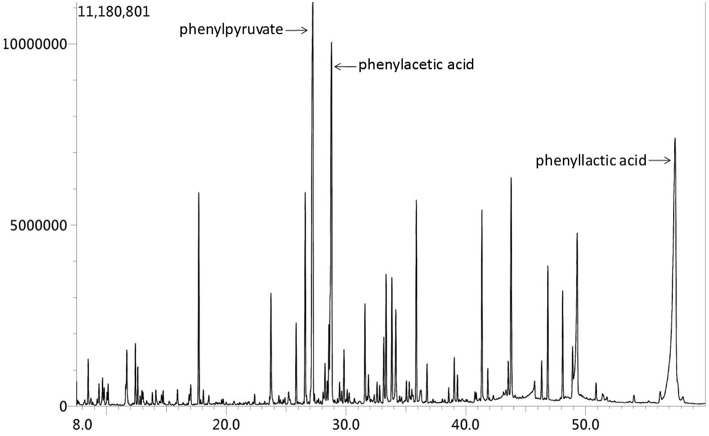
Urine organic acid spectrum showed that phenylacetic acid, phenyllactic acid and Phenylpyruvate increased, suggesting hyperphenylalanine; 2.4-hydroxyphenyllactic acid increased, which may be secondary to liver function damage.

Based on above evidence, the diagnosis of hyperhomocysteinemia (HHcy) and vitamin B12 deficiency was clear. But the etiology of demyelination in white matters required clarification and inherited metabolic-related leukoencephalopathy came out in our mind considering the characteristic findings on head MRI and the positive family history. We instigated urine and plasma amino acid with urine organic acid and plasma acylcarnitine analyses to report the markedly elevated Phe levels (1221.5 μM, normal < 120 μM) and Phe/Tyr ratio (27.45, normal 0.30–2.00) in serum and increased phenyllactic acid and phenylketone acid in urine (Figure 4), consistent with cPKU. We conducted whole exome sequencing (WES) on the patient and his two younger brothers (Figure 2: The fourth and eighth brothers), and the results revealed mental retardation in the patient and his younger brother showing heterozygous mutations in exon12 encoding PAH c.1068C>A and c.740G>T without mutation in the BH4-relating genome, while the youngest brother was a carrier with only c.1068C>A.

Finally, the patient was diagnosed with PKU overlapping HHCy and vitamin B12 deficiency. A previous studies reported that mentally retarded patients with PKU with delayed diagnosis had substantial benefits from a Phe-restricted diet (14, 15). However, the treatment of the patient and his youngest brother was refused by their guardian, due to the financial condition. The patient passed away 4 months post diagnosis (November 2018).

## Discussion

PKU is inherited as an autosomal recessive condition. The gene encoding PAH is located on chromosome 12 and consist of 13 exons, 12 introns, covering a total of 100 kb of genetic data (16). According to the BIOPKU database^1^, more than 1,000 mutations in the gene encoding PAH are known to be associated with PAH deficiency, and the majority of them are missense and result in protein misfolding or impairment of catalytic functions. PKU arises when both alleles are mutated. The severity of PAH deficiency in most cases is determined by the least severe of two PAH mutations. As well as that, those with similar severity may confer a milder phenotype than either of the mutations would do if it acted alone (3). The genotype helps to determine the protein dysfunction, residual PAH activity and, consequently, the metabolic phenotype, i.e., blood phenylalanine levels. But PAH is structurally unstable and susceptible to gene modifiers and non-genetic factors (17–20), therefore, individuals with same genotype may have different metabolic phenotype. In blood, Phe competes with other LNAAs, including tryptophan (Trp) and Tyr, for L-amino acid transporter 1 (LAT1), which is responsible for their uptake into the brain (1). Thus, HPA simultaneously increases the brain Phe levels and reduces other amino acids levels, affecting the neurotransmitter production, protein synthesis, myelin metabolism, and glutamatergic synaptic transmission (21–24), resulting in neuropsychological function impairment. According to the blood Phe level at the time of diagnosis or dietary Phe tolerance, the severity of the disorder varies between patients and, as mentioned above, is classified as MHP, mild HPA-gray zone, mild PKU, moderate PKU, and cPKU. However, previous studies have shown that the inter-individual variations in the kinetics of Phe uptake and metabolism lead to different brain concentrations of the neurotoxin Phe at comparable blood levels (25), which could explain why patients with the same metabolic phenotype may develop different clinical phenotype, especially in terms of cognitive impairment. In conclusion, the severity of clinical phenotype in patients with PAH deficiency is affected by both genotype and metabolic phenotype, in which genotype plays a leading role.

The main characteristic of this case is that the patient showed relatively milder cognitive impairment in the absence of medical intervention, compared to the other six patients in this pedigree who all suffered mental retardation far too serious to live by themselves. Referring to literature, we find that the c.1068C>A is a null mutation (Try>termination codon) on exon 11, which generally causes severe phenotype (26), and the c.740G>T is a missense mutation (Gly>Val) on exon 7. Patients carrying it display mPKU and cPKU phenotypes (27), hence these two mutations genotype may confer a relatively milder phenotype than cPKU. For the patient and his fourth brother, they shared the same genotype with the same mutation sites in chromosome 12 encoding PAH, thereby, the reason why they exhibit phenotypic heterogeneity may be either PAH structure instability or inter-individual kinetics variations of LAT1 on the blood-brain barrier, as mentioned above.

Another important characteristic of our patient is the bilateral diffuse symmetric white matter lesions on brain MRI. It is worthy to note the imaging manifestations of PKU patients because it can indirectly reflect the pathological changes and also show the current Phe concentration of PKU. It is generally accepted that the white matter pathology was likely to reflect hypomyelination in untreated PKUs and intramyelinic edema in early-treated patients (28). However, there are different viewpoints on the targets of elevated Phe in the brain. Some people hold that the direct toxic effect of Phe on oligodendrocytes leads to a dysmyelination (29), others believe that its neurotoxicity is reflected in secondary hypomyelination due to the damage of axonal maturation (30) or neurons (31). Anyhow, the changes witnessed in MRI is thought to be a manifestation and not the initial step of brain injury. Otherwise, the involvement of white matter on MRI found in over 90% of patients were closely related to the present dietary condition and the Phe concentration (32, 33), instead of severity and patterns of neurophysiological alterations. With the decrease in the concentration of Phe after several months by taking treatment, MRI abnormalities show improvement in some patients (34). In our patient, the involvement of the U-fibers and the atrophy in temporal and frontal cortices are consistent with previous reports (28, 35). We considered the restriction in peripheral areas of lesions on DWI (Figures 3C,G) with the normal apparent diffusion coefficient (ADC) map, which suggested that the expansion of the lesion from the occipital lobe to the frontal lobe and from the deep white matter to the subcortical white matter consistently progressed with the disease. The brain CT of his fourth brother showed only slight involvement of bilateral paraventricular white matter, mainly around the posterior horn (Figures 3D,H). Therefore, the patient had relatively severe neuroimaging changes, presenting a gradual deterioration trend compared to his brother, which may be due to the recent elevation of Phe level in his brain and could be indirectly observed by imaging results.

Actually, myelin formation and maintenance is affected by many heterogeneous groups of genetic disorders that include leukodystrophies, inherited vasculopathies, mitochondrial disorders, which all show white matter lesions in imaging. Making the differential diagnosis more challenging, late-onset forms of these disorders exhibit similar clinical manifestations (36). Nowadays, most of these disorders are treatable due to the advent of gene editing, enzyme replacement therapy and hematopoietic stem cells transplantation. Therefore, we summarize 10 leukodystrophies with major prevalence in adulthood in Supplementary Table 2, providing information on the age of onset, underlying genetic mutations, biochemical assays, especially on neuroimaging that aid diagnosis, where available. We also give a brief description of other inherited disorders with white matter involvement in Supplementary Table 3. By doing so, we hope to provide helpful information for timely recognition of adulthood leukodystrophies in a routine clinical setting.

Last but not least, patient in this case was found to have multiple metabolic disorders for vitamin B12 deficiency and HHCy. It's generally known that both HHCy and vitamin B12 deficiency are the risk factors for white matter damage (37, 38) and low vitamin B12 status is thought to be the determinant for HHCy (39). Besides, mild-moderate HHcy [(HCy): 10–100 μM] is an independent risk factor commonly causing cognitive impairment in older adults (40) and a significant trend for an association between severity of dystonia and homocysteine has been reported (41). Hence, we strongly suspect that the sudden deterioration and progressive aggravation of the patient's clinical symptoms may have a correlation with the comprehensive effects of multiple metabolic disorders.

## Conclusion

The majority of the genetic leukoencephalopathy begins in childhood and have been described in detail by the neuropaediatricians. However, the late-onset forms display different clinical and radiological features, at times very far from the classical pediatric description, hence they are placed as intractable rare cases under intensive investigation. Due to the advancement in the MRI technique, it is now possible to frequently detect heritable leukoencephalopathy in adults. Therefore, we recommend neurologists, pediatric neurologists, and radiologists to come up with a set of differential diagnosis and a battery of tests to be performed, in order to standardize the diagnosis methods and treat genetic leukoencephalopathy effectively.

## Data Availability Statement

Publicly available datasets were analyzed in this study. This data can be found here: https://www.ncbi.nlm.nih.gov, http://www.biopku.org/home/home.asp.

## Ethics Statement

The patient had no capacity for consent given his mental status. We have obtained the written informed consent for publication from his guardian.

## Author Contributions

SC collected and interpreted the data and wrote the first draft of this manuscript. MZ and YH participated in literature search and collation. JF and YZ reviewed and revised the manuscript and gave final approval.

### Conflict of Interest

The authors declare that the research was conducted in the absence of any commercial or financial relationships that could be construed as a potential conflict of interest.

## Footnotes

^1^http://www.biopku.org
