# Supplementary material for: Effect of Delayed Diagnosis of Phenylketonuria With Imaging Findings of Bilateral Diffuse Symmetric White Matter Lesions: A Case Report and Literature Review
Source: Front Neurol. 2019 Oct 4;10:1040. doi: 10.3389/fneur.2019.01040 (PMC6788382; doi:10.3389/fneur.2019.01040)
Supplement: Supplementary file 1 [file Table_1.DOCX]

**Supplementary Table 1. A summary of adult-onset or late-diagnosed patients with PKU.**

| **Country** | **Sex** | **Age of onset (y)** | **Clinic symptoms** | **Neuroimaging** | **Phe level at diagnosis**  **(µM)** | **Gene mutation** | **Prognosis** | **Family history associated with PKU** |
| --- | --- | --- | --- | --- | --- | --- | --- | --- |
| Japan, 1993 (42) | M | 32 | Blurred vision, spastic gait disturbance, IQ=68 | MRI T2-weighted sequences: widespread high-intensity areas in the deep white matter especially in bilateral occipital lobes. | 1663 | NA | PHE-restricted diet, no  recovery | NA |
| Germany, 2000 (43) | F | 45 | Mild spastic tetraparesis, ataxia, tremor, severe concentration and anamnestic problems, cognitive deceleration | MRI: severe white-matter abnormalities in parieto-occipital, frontal and subcortical areas. | 882 | R408W/R68S | PHE-restricted diet, almost complete recovery | NA |
| USA, 2001 (44) | F | 53 | Slowly progressive spastic paraparesis and dementia, IQ=108 | Normal (MRI) | 2153 | R158Q/IVS12+1G >A | Protein restricted diet, slight improvement | One younger brother with PKU |
| France, 2009 (45) | M | 54 | Tetraparesis, cognitive impairment, and an unusual body odour | MRI FLAIR sequences: symmetrical hyperintense in the deep cerebral white matter in bilateral parietal-occipital regions. | 1140 | I65T/R252W | PHE-restricted diet, no recovery | N |
| UK,  2014 (46) | M | 40 | Tonic clonic seizures, epilepsy and learning difficulties | Normal (CT) | 1451 | NA | PHE-restricted diet | N |
|  | M | 42 | Severe learning difficulties | NA | 1670 | NA | NA |  |
| Italy, 2014 (47) | F | 46 | mild learning difficulties, acute rapidly progressive dementia, visual impairment, prosopagnosia and parkinsonism | MRI: Diffuse bihemispheric white matter hyperintensity, mild cortical atrophy.  MRS: decreased NAA/Cr ratio. | 947 | IVS10-11G>A/IVS4+4A>G | Phe-restricted diet, rapid improvement | One older sister with PKU |
| France, 2014 (48) | F | 47 | early-onset parkinsonism, tremor, epilepsy, mental retardation | NA | 2370 | IVS4+5G>T/P281L | No specific diet | NA |
| France, 2015 (49) | F | 20 | mental retardation, light skin and hair, IQ<65 | MRI: bilateral and symmetric periventricular leukoencephalopathy. | 1687 | Homozygote mutation | No specific diet | Parents are first cousins |
| Turkey, 2016 (50) | M | 59 | rapidly progressive dementia, blurred vision, and parkinsonism | MRI T2-weighted sequences: bilateral diffuse hyperintense lesions in parietal and occipital white matter. | 1075 | NA | PHE-restricted diet, significantly improved | NA |
| China, 2018 (51) | M | 29 | progressive dementia and muscular weakness | MRI T2-weighted sequences: symmetrical high-intensity of the deep cerebral white matter in bilateral occipital and parietal regions. | 966.67 | G247V/R243Q | Protein restricted diet, partly improved | Older sister with mental retardation as a child |
| China, 2018 (52) | M | 21 | visual-spatial disorders and personality changes | MRI FLAIR T2-weighted sequences: hyperintensities in periventricular and subcortical white matter. | 694 | p.G247R/p.Y204C | Protein restricted diet, back to normal | N |
| Our case | M | 60 | Mental retardation, spastic tetraparesis, tremor, visual-spatial disorders and personality changes | MRI: Diffuse bihemispheric white matter hyperintensity, mild cortical atrophy. | 1221.5 | p.Y356*/p.G247V | No specific diet | seen in FIGURE 2 |

M: male; F: female; N: negative; NA: not available; MRS: MR spectroscopy; NAA/Cr ratio: N-acetyl-aspartate/ creatine ratio; IQ: intelligence quotient.
