# Supplementary material for: Effect of Delayed Diagnosis of Phenylketonuria With Imaging Findings of Bilateral Diffuse Symmetric White Matter Lesions: A Case Report and Literature Review
Source: Front Neurol. 2019 Oct 4;10:1040. doi: 10.3389/fneur.2019.01040 (PMC6788382; doi:10.3389/fneur.2019.01040)
Supplement: Supplementary file 2 [file Table_2.DOCX]

**Supplementary Table 2. Inherited leukodystrophies with major prevalence and/or onset in adulthood.**

| **Disease** | **Age of onset** | **Gene(s) and coding protein(s)** | **Biochemical findings** | **Neuroimaging** |
| --- | --- | --- | --- | --- |
| Alexander disease (AxD) (53) | Childhood to adulthood | GFAP: glial fibrillary acidic protein (GFAP) | Elevated glial fibrillary acidic protein in cerebrospinal fluid;  Pathology: Rosenthal fibres | Extensive cerebral white matter changes with frontal predominance; a periventricular rim with high signal on T1-weighted images and low signal on T2-weighted images; abnormalities of basal ganglia and thalami; atrophy and signal intensity changes in the medulla oblongata and upper cervical spinal cord; contrast enhancement of particular gray and white matter structures |
| X-Linked adrenoleukodystrophy (X-ALD) (54) | Adrenomyeloneuropathy (AMN): 14-60 years;  Adulthood cerebral adrenoleukodystrophy (ACALD): ≥21 years | ABCD1: peroxisomal membrane protein ATP-binding cassette sub-family D member 1 (ALDP) | Elevated saturated very long chain fatty acids in serum | Occipital regions white matter changes progressing anteriorly, with early involvement of the splenium of the corpus callosum and posterior limbs of the internal capsule.  AMN: posterior limbs of the internal capsules along with cerebellar and brainstem white matter involved, spinal cord atrophy may develop.  ACALD: periphery contrast enhancement |
| Adult polyglucosan  body disease (APBD) (55) | 50-60 years | GBE1: glycogen-branching enzyme | Polyglucosan accumulation in skin and nerves | Hyperintense white matter abnormalities on T2 sequences predominantly in the periventricular regions, the posterior limb of the internal capsule, the external capsule, and the pyramidal tracts and medial lemniscus of the pons and medulla; the medulla and spine atrophy; sparing of the U fibres and corpus callosum. |
| Autosomal dominant adult-onset demyelinating leukodystrophy (ADLD) (56) | 50-60 years | LMNB1  Duplication: lamin B1 protein overexpression | None | Diffuse white matter T2 hyperintensities involving the frontal lobe, parietal lobe and mainly middle cerebellar peduncle; brainstem and corpus callosum atrophy. |
| Cerebrotendinous xanthomatosis (CTX) (57) | Childhood to adulthood | CYP27A1: mitochondrial enzyme sterol 26-hydroxylase | Elevated serum cholestanol and bile alcohols; reduced mitochondrial sterol 26-hydroxylase activity in leukocytes | MRI: High signal intensity within the cerebellar white matter and low signal intensity in the dentate nucleus on T2-weighted imaging. Non-specific deep periventricular white matter change with sparing of U fibres and corpus callosum. |
| Globoid cell leukodystrophy (GLD; also known as Krabbe disease) (58) | Childhood to adulthood | GALC | Galactocerebrosidase deficiency in leukocytes or fibroblasts | CT: Thalamus and basal ganglia hyperdense  MRI: Posterior predominant white matter changes, with sparing of the U fibres and involvement of the splenium of the corpus callosum; tigroid pattern; T2 hyperintense and enhancement of corticospinal tracts. |
| Hereditary diffuse leukoencephalopathy with spheroids (HDLS) (59) | 40–70 years | CSF1R: the tyrosine kinase domain of CSF-1R | None | MRI: non-enhancing, symmetrical white matter T2 hyperintensity with frontal prominence , spreading out from the periventricular and deep white matter into the subcortical areas, posterior limb of the internal capsule and the pyramidal tracts of the brainstem; corpus callosum involved. |
| Leukoencephalopathy with brainstem and spinal cord involvement and lactate elevation (LBSL) (60) | Childhood to adulthood | DARS2: mitochondrial aspartyl-tRNA synthetase | None | MRI: Inhomogeneous cerebral white matter abnormalities sparing the U-fibers and strikingly selective tract(entire length of pyramidal tracts and sensory tracts) involvement. Cerebellar connections are selectively involved. The consistent involvement of the intraparenchymal trajectories of the trigeminal nerve and mesencephalic trigeminal tracts is remarkable. |
| Leukoencephalopathy with vanishing white matter (VWMD) (61) | Childhood to adulthood | EIF2B1-5: eukaryotic translation initiation factor eIF2B | None | An abnormal signal of all or almost all cerebral white matter with associated cystic change; relatively sparing of U-fibres; later: white matter may vanish leaving a ventricular wall and cortex. |
| Metachromatic leukodystrophy (MLD) (62) | Childhood to adulthood | ARS: arylsulfatase A(ASA)  PSAP: prosaposin | Decreased arylsulfatase A activity, elevated urinary sulfatides | MRI: Widespread symmetrical confluent white matter change with periventricular, frontal predominance; tigroid pattern; early involvement of corpus callosum and sparing of subcortical U fibres. |

Tigroid pattern: a pattern of radiating stripes with bands of normal signal intensity within the sheet-like white matter abnormality cerebral white matter on MR images in lysosomal storage disorders. (63)
