# Supplementary material for: Effect of Delayed Diagnosis of Phenylketonuria With Imaging Findings of Bilateral Diffuse Symmetric White Matter Lesions: A Case Report and Literature Review
Source: Front Neurol. 2019 Oct 4;10:1040. doi: 10.3389/fneur.2019.01040 (PMC6788382; doi:10.3389/fneur.2019.01040)
Supplement: Supplementary file 3 [file Table_3.DOCX]

**Supplementary Table 3. Other heritable diseases with prominent white matter involvement.**

| Inherited vasculopathies | | | | |  |
| --- | --- | --- | --- | --- | --- |
| **Disease** | **Age of onset** | **Gene(s)** | **Clinical signs** | | **Neuroimaging** |
| Cerebral autosomal dominant arteriopathy with subcortical infarcts and leukoencephalopathy (CADASIL) (64) | Child-hood to old age | NOTCH3: a single-pass  transmembrane receptor in arterial smooth-muscle cells | migraine with aura, recurrent subcortical ischaemic events, mood disturbances, apathy, and cognitive impairment | | MRI T2-weighted imaging or FLAIR : bilateral diffuse hyperintensity in white matter, mostly symmetrical, and mostly occur in the external capsule and the anterior part of the temporal lobes. |
| Cerebral autosomal recessive arteriopathy with subcortical infarcts and leukoencephalopathy (CARASIL) (65) | 10 - 50 years | HTRA1: HtrA serine peptidase/protease 1 (HTRA1) | Spasticity gait disturbance, stroke-like episodes, mood changes (apathy and irritability), pseudobulbar palsy, cognitive dysfunction, alopecia and spondylosis | | Extended white matter lesions and external capsule lesions resembling that of CADASIL. U-fibers relatively preservation. |
| **Mitochondrial disorders** (66) | | | | | |
| Classification: Leigh syndrome, Kearns-Sayre syndrome (KSS), mitochondrial encephalomyopathy lactic acidosis and stroke like episodes (MELAS), mitochondrial neuro-gastro-intestinal encephalomyopathy (MNGIE), Leber’s hereditary optic neuropathy (LHON) | | | | Suggestive MRI findings: small cyst-like lesions in abnormal white matter, involvement of both cerebral and cerebellar white matter, combination of a leukoencephalopathy and bilateral basal ganglia lesions. | |
